# A Computational Model Incorporating Neural Stem Cell Dynamics Reproduces Glioma Incidence across the Lifespan in the Human Population

**DOI:** 10.1371/journal.pone.0111219

**Published:** 2014-11-19

**Authors:** Roman Bauer, Marcus Kaiser, Elizabeth Stoll

**Affiliations:** 1 Interdisciplinary Computing and Complex BioSystems Research Group (ICOS), School of Computing Science, Newcastle University, Newcastle upon Tyne, Tyne and Wear, United Kingdom; 2 Institute of Neuroscience, Newcastle University, Newcastle upon Tyne, Tyne and Wear, United Kingdom; National University of Singapore, Singapore

## Abstract

Glioma is the most common form of primary brain tumor. Demographically, the risk of occurrence increases until old age. Here we present a novel computational model to reproduce the probability of glioma incidence across the lifespan. Previous mathematical models explaining glioma incidence are framed in a rather abstract way, and do not directly relate to empirical findings. To decrease this gap between theory and experimental observations, we incorporate recent data on cellular and molecular factors underlying gliomagenesis. Since evidence implicates the adult neural stem cell as the likely cell-of-origin of glioma, we have incorporated empirically-determined estimates of neural stem cell number, cell division rate, mutation rate and oncogenic potential into our model. We demonstrate that our model yields results which match actual demographic data in the human population. In particular, this model accounts for the observed peak incidence of glioma at approximately 80 years of age, without the need to assert differential susceptibility throughout the population. Overall, our model supports the hypothesis that glioma is caused by randomly-occurring oncogenic mutations within the neural stem cell population. Based on this model, we assess the influence of the (experimentally indicated) decrease in the number of neural stem cells and increase of cell division rate during aging. Our model provides multiple testable predictions, and suggests that different temporal sequences of oncogenic mutations can lead to tumorigenesis. Finally, we conclude that four or five oncogenic mutations are sufficient for the formation of glioma.

## Introduction

Glioma is the most common form of primary brain tumor [1]. Glioma commonly manifests itself as a high-grade tumor called glioblastoma, a highly malignant and invasive tumor with median patient survival of 12 months from diagnosis; lower-grade gliomas increase in malignancy over time, with associated increases in mortality [2].

The cellular mechanisms giving rise to glioma are subject to intense research. The incidence of glioma is not significantly affected by environmental factors such as UV light and carcinogen exposure, due to the protective influence of the thick skull and the blood-brain barrier. In addition, there are no known heritable factors in the risk of glioma occurrence. These tumors appear to arise idiopathically in a random manner throughout the population [3]. Hence, glioma formation is an ideal test-case for investigating how fundamental mechanisms on the single-cell level give rise to cancer.

Increasing age is strongly associated with higher incidence and increased malignant grade for all grades and types of glioma [4], [5]. Age is in fact the single most robust factor influencing glioma incidence, malignancy, and patient survival [1], [2], [4]. Insights into changes that occur in the aging brain and the cells that originate the tumor are therefore essential for understanding this increased risk of oncogenic transformation and tumorigenesis.

The putative cell-of-origin of glioma is the neural stem cell (NSC), which normally gives rise to new neurons and glial cells in the adult brain. Experimentally causing oncogenic mutations in this lineage leads to the formation of malignant tumors [6]–[8], and gliomas cluster near germinal centers of the brain [9]. Proliferative cells within the tumor share immunomarkers with NSCs [10], [11]. NSCs already exist in a proliferative state, are capable of differentiating into glial cell types, and can migrate through tissue [12], [13]. Transplantation of oncogenically-transformed mouse neural stem cells into syngeneic mice reliably leads to the formation of a tumor which recapitulates the proliferative and invasive phenotype of human glioma [14], [15]. Together, these studies strongly implicate the neural stem cell as the most likely cell-of-origin of glioma. In this report we show that modeling the accumulation of random mutations during cell division in this stem cell population can predict glioma incidence across the lifespan in the human population. In particular, we propose a model that accounts for differential weightage and temporal ordering of oncogenic mutations.

## Materials and Methods

The model includes empirical data collected through literature review. The mutation frequency was taken directly from a published estimate and assumed to be constant across the lifespan [16]. A small subset of mutations were deemed to have oncogenic potential in this cellular compartment while all other mutations are assumed to be neutral for this cancer type [17]. In this approach, we used the Poisson-approximation of a binomial distribution for computing the probabilities to have x oncogenic mutations. First we compute the expected number of genetic mutations a cell has had at a certain age, and based on that then compute the probability of having x oncogenic mutations. The mutation rate is therefore independent of whether or not the gene is oncogenic.

**Figure 1:**
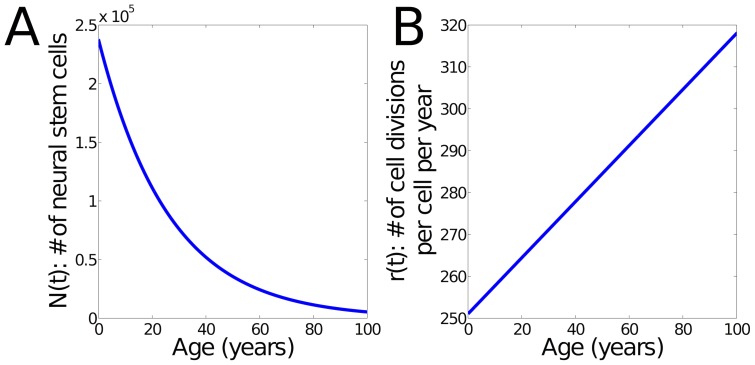
Modeled number and cell division rate of NSCs. **(A)** Number of NSCs during aging. The initial number of cells was estimated based on [18]. The number of NSCs is given by 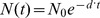 using 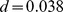. **(B)** Modeled cell division rate over time. As shown in [13], NSCs increase their rate during aging. We have approximated this behavior using a linear interpolation from 251 to 318 divisions per cell and year.

The exponentially decreasing number of neural stem cells was calculated across the lifespan based on the published data for human tissue [18]. Results of electron microscopy-based characterization is shown in Figure 3 of [18], which used 200 micron thick sections. Results of immunohistochemistry-based characterization are shown in Figure 1 of [18], which used 30 micron thick sections. These data are in agreement - 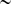
144 cells per 200 micron-thick section (averaging the two locations described above) and 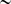
22 DCX+ cells per 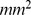 in a 30 micron-thick section (estimated from the graph in Figure 1r of [18]). These data both yield approximately 720 DCX+ cells per 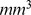. To estimate KI67+ proliferative cells, not DCX+ cells, we multiplied the values for KI67+ cells from the relevant graph (Figure 1s of [18]) by 33, just as we multiplied the values for the DCX+ cells from the other graph (Figure 1r of [18]) by 33. This provides values per 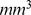. In agreement with the data presented in Figures 1 and 3 of [18], their Figure 2c shows that the tract is 1 mm × 1 mm wide. It is also 10 mm long (the scale bar represents 500 microns). So the number of KI67+ cells per 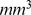 is multiplied again by 10 to estimate the total number of KI67+ cells. The graph of KI67+ cells at each time point was then extrapolated to estimate this population across the entire lifespan. Overall, we computed the number of NSCs at birth to be 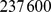, which was used as the initial value of the modeled number of NSCs during aging (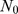).

**Figure 2:**
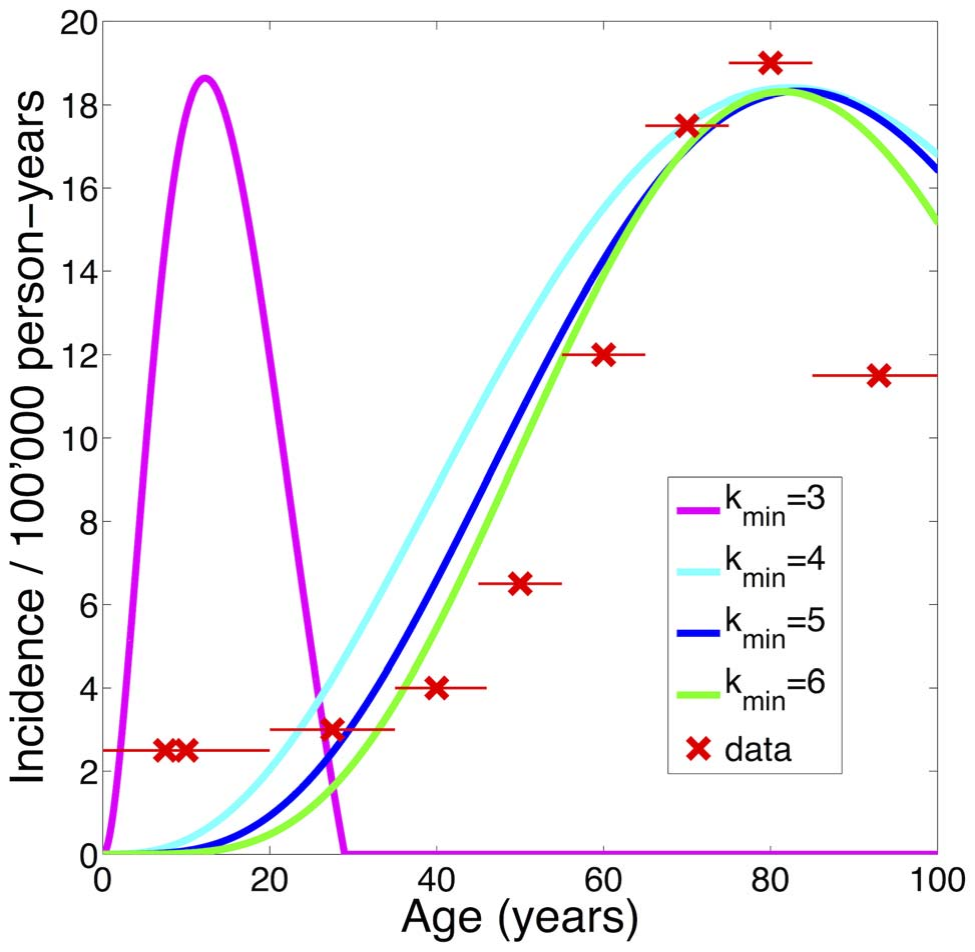
Influence of 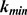 on location of peak incidence. Representative incidence curves for 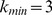 (magenta), 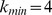 (cyan), 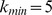 (blue) and 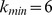 (green). Only for 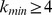 can the condition of peak incidence at approximately 80 years be fulfilled. Incidence curves generated by the model for 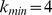, 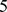 and 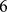 are in accordance with the demographic data from [1] (red crosses: mean incidence of age groups, red lines: spans of age groups), with 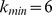 yielding the best fit. Confidence intervals are shown in Fig. S1.

**Figure 3:**
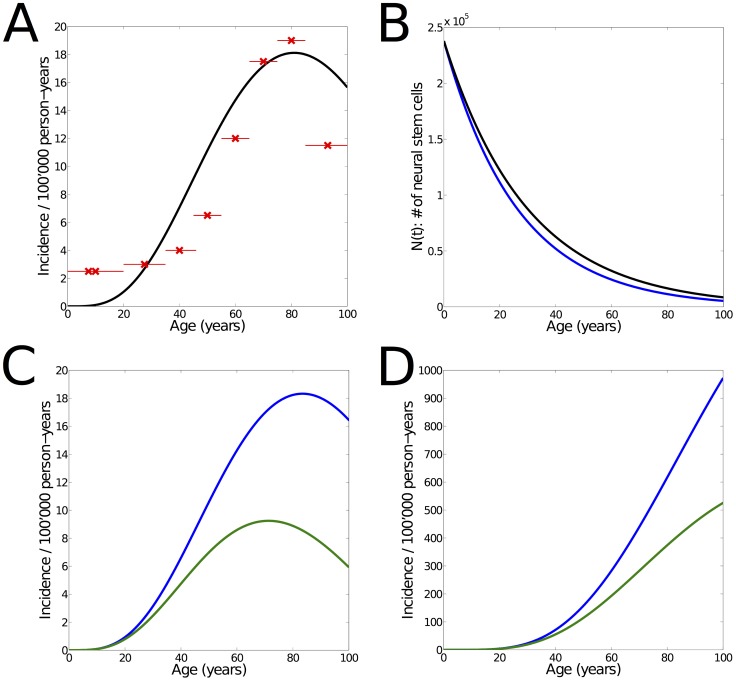
Effect of increasing cell division rate. **(A)** Modeled incidence of glioma (green) under constant cell division rate (
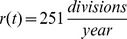
). Model parameters 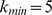, 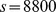 and 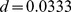 were used in order to match with the demographic data (red crosses: mean incidence of age groups, red lines: spans of age groups). The increasing proliferation rate of NSCs is therefore not a necessary condition for the incidence curve to match the demographic data, since similar results are obtained after changes in the model parameters 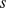 and 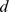. **(B)** Number of NSCs over time, as used for the incidence curve shown in (A) (black) and for the scenario where cell division rate increases linearly (Fig. 2, blue). Small changes in the number of NSCs over time are sufficient to make up for the constant cell division rate. It remains an empirical question which estimates of 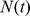 and 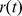 are correct in the adult human, since these are extrapolated from the model, the young human, and the aging rodent. **(C)** Incidence of glioma as derived from our model, for increasing (blue) and constant (green) cell division rate during aging. Model parameters are the same (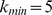, 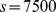, 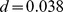). The green curve is the predicted incidence by the model if the proliferation rate was constant, and so leads an estimate of the net effect of the increase. Overall, our model suggests that the increase in cell-cycle re-entry substantially increases glioma formation. **(D)** Prevalence of glioma for increasing (blue) and constant (green) cell division rate. As shown in Fig. S2, the results are qualitatively confirmed also for 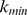 = 4.

The cell division rate was calculated in NSCs derived from the young adult and aged adult mouse brain [13]. The number of cell divisions in a given time was calculated from live-cell time-lapse imaging over a 48 hour period. Actively-cycling young adult NSCs divided 1.37 times in 48 hours while actively-cycling aged adult NSCs divided 1.74 times in 48 hours. Adjusted for time, actively-cycling young adult NSCs divide 251 times per year while actively-cycling aged adult NSCs divide 318 times per year. For the estimate that is incorporated in the model, we have used a linear interpolation between these two numbers across the human lifespan. These estimates were assumed relevant for the population of NSCs in the adult human brain (Fig. 1).

The model was implemented in MATLAB (Mathworks Inc.). A time step dt of 0.001 years was used for calculating the prevalence. The computation of the incidence was done by computing the numerical differential of the prevalence over time. Bootstrapping was used to compute the 95 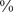 confidence interval of the incidence, as shown in Fig. S1. 1000 bootstrap samples of size 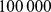 were computed.

Two of the model parameters (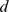 describing exponential decrease of NSCs with time and 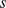 included in Eq. 5) were not assessed from experimental findings. Depending on 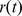 and 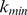, different incidence curves are obtained (i.e. the absolute values and the position of the curve peak were different). We have adapted 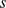 and 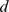 for the different scenarios, in order for the incidence curve to match with the demographic data [1]. A match could only be obtained for 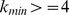. In Fig. 2, 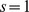 and 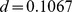 were used for the incidence curve based on 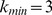, while for 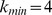 we used 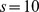 and 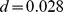. For the simulations using 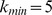 and 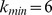 we set 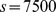, 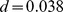 and 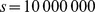, 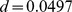, respectively.

## Results

To create our model, we included empirical data representing age-related changes in neural stem cell number and behavior. A population of neural stem cells is present in the human brain at birth but declines exponentially thereafter [18]. Experiments in rodents demonstrate that the exponential decline in neural stem cell number continues across the lifespan [13], [19]. This depletion of the stem cell population is due to cell death and terminal differentiation. We have therefore approximated the size of this cell population (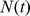) with an exponential interpolation of the data from the human brain. Further experiments have demonstrated that the remaining population of NSCs in the aged brain have dysregulated cell cycle kinetics [13]. Individual remaining stem cells have an increased likelihood of re-entering the cell cycle, resulting in an increased number of cell divisions in a given period of time (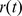). We have approximated this behavior using a linear interpolation. Our model incorporates these empirically-determined changes in neural stem cell number and behavior (Fig. 1).

NSCs accumulate mutations in every cell cycle. The process of genome replication during cell division is imperfect, as a certain number of mutations occur and some of these mutations will remain unrepaired. The number of mutations incurred during a single cell division has been estimated [16]. According to their assessment, we denote by 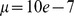 the probability for a gene in the coding region to mutate due to a single cell division. No single mutation leads to oncogenesis, so multiple hits are necessary for complete oncogenic transformation [20], [21]. Cancer is characterized by a number of cellular changes, including loss of cell cycle control, self-sufficiency in growth factor signaling, resistance to anti-growth signals, escape from apoptosis, invasion and neovascularization [22]. When Hanahan and Weinberg first described these hallmarks of cancer, they proposed that approximately six mutations would be required to dysregulate all six of these cellular activities [22]. Yet now researchers appreciate that mutation of a single multi-functional protein can predispose alterations to multiple cellular activities [14], [23]. Since the cell is dependent upon semi-redundant regulatory pathways to control cell cycle progression and other activities [20], loss of one major tumor suppressor is not sufficient to create a tumor [21] and multiple regulators must be disrupted to achieve oncogenic transformation [14], [24]. Of the 18 440 (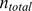) protein-encoding genes in the human, 522 have a causal role in human cancer and 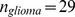 of these (Table S1) have a demonstrated role in promoting gliomagenesis [17]. We assessed how many mutations in this set of oncogenes are required to achieve tumor formation. Based on this minimum number of mutations (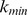), our model computes the total probability for a single NSC to become oncogenically transformed. This integrative probability is calculated by summing up the individual probabilities according to the following equation:
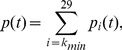
(1)where 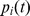 denotes the probability for 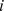 oncogenic mutations to have occurred at time t. We have estimated 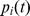 using the experimentally assessed parameters 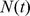, 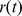 and 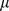. Based on the number of protein-coding and gliomagenesis-relevant genes, the probability for any one of the 29 oncogenes to become mutated from cell division is given by 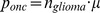. Assuming that any gene mutates with equal probability, the occurrence of oncogenic mutations can be approximated by the binomial distribution. It follows that 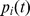 is given by:
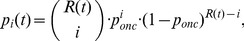
(2)where 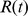 is the number of cell divisions a NSC has undergone until time 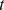. It is computed by integrating the cell division rate 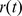 across the age span until time t.

Given that 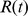 and 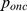 take sufficiently high (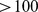) and low (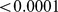) values respectively, the Poisson distribution is well-suited as an approximation for this otherwise computationally very demanding formula:
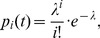
(3)with 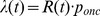. The temporal sequence of oncogene mutations has been shown to be an important factor in tumor formation [25], [26], and so we have also accounted for it in our model. Given that there are 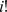 possibilities for 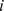 mutations to occur, Eq. 3 becomes:
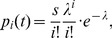
(4)where the scalar value 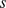 represents the number of specific mutational sequences necessary for oncogenic transformation. For 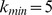, we find 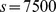 to be an appropriate value in order for the incidence curve to be in numerical accordance with the demographic data (Fig. 2). This means that on average 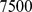 different sequences of mutations exist (for the different scenarios, i.e. 5, 6,…, 29 oncogenes affected), which can ultimately lead to oncogenic transformation.

The probability for a single cell to become oncogenically transformed is denoted by 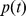. Accordingly, the probability for glioma formation overall is proportional to the probability that at least one of all the NSC becomes transformed:
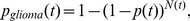
(5)where 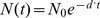 is the estimated number of NSCs at time t. Hence, the parameter 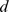 describes the decay of the NSC population over time, and so is in principle directly relatable to empirical data. We have adapted 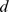 such that the resulting incidence curve matches the demographic data, while being qualitatively in accordance with experimental findings in the mouse [13], [19].

The prevalence of glioma is then proportional to 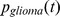. Since the units from the demographic datasets are with respect to 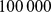 person-years, we compute the prevalence by multiplying 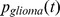 by 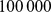. From this, the incidence is computed by calculating the derivative. Since there are various time-varying parameters in the model, an analytical differentiation comprises a too extensive formula. We therefore assess the incidence numerically. The obtained incidence curve is shown in Fig. 2 and resembles the demographic data.

The actual incidence of glioma across age demographics has been documented by The Central Brain Tumor Registry of the United States [1]. We have used these published data to provide a fit for the incidence and prevalence of glioma across the lifespan (Fig. 2). The model parameters 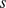 and 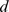 were adapted in order to match with these incidence rates. The incidence curves obtained from our model for 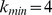, 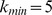 or 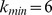 resemble these demographic data. Also for 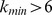 is it possible to achieve agreement, and so our model yields a lower bound for the number of mutations required for oncogenic transformation. However, with increasing 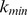 the model parameters 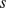 and 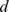 need to change too. In particular, the parameter 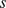 strongly increases. For 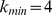, 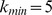 and 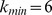 we find 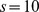, 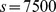 and 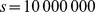 to be well-suited, respectively.

The biological meaning of parameter 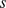 in Eq. 4 is twofold. It captures that different oncogenes can yield the same transformation hallmarks [6], [24], and so multiple sequences of the same length could give rise to glioma. Additionally, 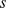 accounts for the possibility that different temporal sequences of the same oncogenes could lead to glioma formation. In the classical multistage model, there is only one temporal order that can achieve transformation. Importantly, since 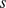 denotes an average number of mutations, it could be different for different sequence lengths 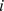. With increasing 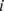, 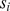 can grow exponentially because of the factorials in the denominator of Eq. 4. For simplification and due to lack of detailed empirical knowledge, we chose to use the same 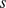 for all sequence lengths.

Since no studies in the human have directly demonstrated increased cell division in NSCs, we have created a related model that assumes no age-related changes in cell division rate, cell cycle length or likelihood to re-enter cell cycle. This adjusted model yields the same results in glioma incidence and required mutation number if the exponential decrease in proliferative cell number is adjusted accordingly (Fig. 3A). This age-related change is therefore not a necessary condition of the model. Future labelling studies of the proliferative cell population in the human brain will help to evaluate the relative accuracy of these two models. Interestingly, the model quantifies the net effect of an increasing cell division rate while the other parameters are the same (Fig. 3CD). These results suggest that this increase of cell division rate almost doubles the occurrence of glioma.

## Discussion

Mathematical modeling has been used to create predictions regarding the growth of tumors [27], [28] and response of individual tumors to surgical resection or radiotherapy [29], [30]. The incidence of tumors in a human population has also been modeled [31], [32]. However these models of cancer incidence did not employ empirical measures of age-related changes in cellular dynamics, nor did they incorporate experimental knowledge on glioma-related proto-oncogenes. Here we present a model to predict the probability of glioma incidence across the lifespan based on neural stem cell dynamics in the individual organism.

We find that a simple model using recent estimates of biological parameters on the single-cell level can account for demographic observations. Along these lines, we provide a modified and extended version of the well-established Armitage-Doll model [31]. In contrast to this classical approach, we do not restrict our model to a specific number of oncogenic mutations. Instead, we account for all the numbers of oncogenic mutations that possibly can occur (i.e. mutations of 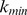 to 29 oncogenes, see Eq. 1). Our model therefore does not rely on the (experimentally unsupported) assumption of the classical Armitage-Doll model that only a specific number of oncogenes must be mutated for oncogenic transformation.

Since the parameters of our model have a direct biological meaning, further biological data can be incorporated and predictions can be made. For example, previous theories have yielded various estimates for the minimal number of oncogenic mutations required for carcinogenesis [33]–[35]. Notably, we come to the conclusion that a minimum of 4 or 5 oncogenic mutations is sufficient for tumorigenesis, in contrast to 6-7 mutations as implicated by the classical Armitage-Doll model [31] and as predicted by Hanahan and Weinberg [22]. 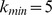 is higher than experimental results which demonstrate that NSCs can be oncogenically transformed successfully with only three oncogenic mutations specifically affecting the PTEN, p53 and Rb pathways [14], [24], [36]. However, many human gliomas regardless of grade demonstrate 5 mutations, namely affecting EGFR, PTEN, 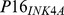, TP53 and MDM2 [3]. Therefore our model is in line with empirical studies on the number of mutations required to achieve oncogenic transformation. Many mutations affecting tumor suppressor pathways will cause a cell to undergo senescence, slowing the cell division rate and increasing the likelihood of apoptosis. Very few sequences of mutation are likely to bypass this protective response. So it is easy to imagine that few scenarios (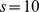) are compatible with a low number of mutations achieving oncogenic transformation (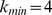), while more scenarios (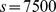) can achieve oncogenic transformation with a larger number of mutations (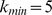). Considering that different oncogenic mutations yield the same hallmark, and that multiple temporal sequences of the same mutations could yield the same result, we find 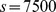 more plausible than 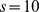. This model therefore supports the conclusion that five oncogenic mutations are sufficient to achieve oncogenic transformation and initiate gliomagenesis.

Our model accounts for the possibility that some oncogenes, due to more interactions, play a more central role than others [37]. Therefore, fewer mutations of such hub genes might be sufficient for the formation of glioma. It is possible that altered function of such hub genes could lead to genomic instability and increased mutation rate. However, one assumption in our model is the stable accumulation of mutations in every cell cycle. While this number of mutations have been estimated in proliferative cell types [16], this rate may indeed depend on prior changes. With age, the genome becomes more unstable due to shortened telomeres, increased mutation load and chromosomal abnormalities [38]. All of these changes could increase the likelihood of mutations or disrupt the efficacy of repair mechanisms. The net mutations incurred during each division may therefore increase with age. However any age-related changes to the mutation rate depending on prior mutation load have not been empirically determined so we were unable to incorporate this age-related factor into our calculations. We have therefore estimated that the mutation rate remains constant across the lifespan.

However our model does allow us to incorporate different weightage for mutations, i.e. that some mutations are less likely to co-exist than others, as has been established by the Cancer Genome Atlas effort (http://cancergenome.nih.gov/) [21], [25]. In Eq. 4 the denominator increases much faster than the nominator with the length of the modeled sequence of oncogenes, and so long sequences are unlikely to occur. Hence, mutational combinations that are included only in the long sequences are unlikely to co-exist overall.

In light of evidence that a temporal sequence of mutations may be crucial in tumorigenesis [25], [26], it is notable that our model considers variation in the number and order of oncogenic mutations needed to invoke glioma formation. Our model thus usefully explores the relationship between these experimentally tractable variables, particularly 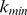, 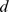, 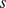, 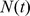 and 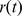.

Similar to previous researchers [32], we have included an age-related decline in the number of proliferative cells, which is responsible for the characteristic peak of the incidence at 80 years. In contrast to their linear decrease, we model an exponential decrease of the proliferative pool which matches better with experimental findings in this cell population [13], [18], [19]. In addition, we employ empirically-derived results to estimate cell cycle length [12], the mutation rate during each cell cycle [16] and the fraction of genes that promote oncogenic transformation upon mutation ([17] and Table S1). Together, these data can be used to predict the age-associated incidence of glioma in the human population [1] without the need to assert differential susceptibility throughout the population which is not supported by biological evidence [39].

It is possible that other cell types besides the neural stem cell give rise to glioma. One recent study demonstrated that mature cells such as neurons can be forced to undergo oncogenic transformation using cell-specific targeting of two major tumor suppressor pathways [36], however it is not clear that such mutations could randomly occur in a post-mitotic cell population. Alternatively, glial progenitor cells within the white matter have been proposed to be the true glioma cell-of-origin [40], [41]. Empirical data on these cells are scarcer, so we are currently unable to estimate the size of this population and the rate of glial progenitor cell division across the lifespan (key variables for implementing this model). Future studies may help to address whether the cell cycle kinetics of this population can also predict actual glioma incidence in the human population. Variability in the cellular origin as well as the underlying genetic lesions of glioma could in part explain the extraordinary heterogeneity in this tumor type. Yet the evidence most strongly implicates the multi-potent neural stem cell as the most likely cell of origin, so we have focused on this cell type in our model.

There is evidence to suggest the molecular pathogenesis of high-grade gliomas (presenting as primary glioblastoma) is different to that of low-grade gliomas (presenting as grade II-III astrocytoma or oligodendroglioma, often progressing to secondary glioblastoma). These two types of brain tumor have different genetic and epigenetic profiles, with different initiating mutations [42]. In the future, this model could be adapted to include such different constraints on molecular pathogenesis to distinguish between the incidence rates of low-grade and high-grade glioma.

Overall, we provide a model that uses experimentally obtained parameters on neural stem cell proliferation and yields results which match with actual demographic data in the human population. We demonstrate the consistency of our model which incorporates estimates of neural stem cell number, cell division rate, mutation rate and number of oncogenes. Importantly, our model supports the hypothesis that glioma is caused by randomly occurring oncogenic mutations within the neural stem cell population of the adult brain.

## Supporting Information

Figure S1
**Confidence intervals for modeled incidence.** 95 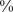 confidence intervals (shaded) for the modeled incidence rates during aging, as computed by bootstrapping. The modeled incidence curve (blue line) is the same as shown in Fig. 2 using **(A)** 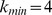, 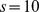 and 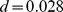, **(B)** 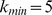, 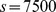 and 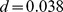 and **(C)** 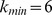, 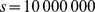 and 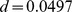.(PDF)

Figure S2
**Effect of increasing cell division rate for scenario with** 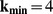. **(A)** Modeled incidence of glioma (black) under constant cell division rate (
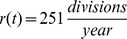
). Model parameters 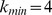, 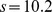 and 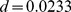 were used in order to match with the demographic data (red crosses: mean incidence of age groups, red lines: spans of age groups). The increasing proliferation rate of NSCs is therefore not a necessary condition for the incidence curve to match the demographic data, since similar results are obtained after changes in the model parameters 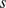 and 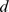. **(B)** Number of NSCs over time, as used for the incidence curve shown in (A) (black) and for the scenario where cell division rate increases linearly (Fig. 2, cyan). Small changes in the number of NSCs over time are sufficient to make up for the constant cell division rate. It remains an empirical question which estimates of 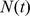 and 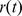 are correct in the adult human, since these are extrapolated from the model, the young human, and the aging rodent. **(C)** Incidence of glioma as derived from our model, for increasing (cyan) and constant (green) cell division rate during aging. Model parameters are the same (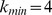, 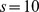, 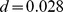). The green curve is the predicted incidence by the model if the proliferation rate was constant, and so leads an estimate of the net effect of the increase. Overall, as for 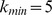 our model suggests that the increase in cell-cycle re-entry substantially increases glioma formation. **(D)** Prevalence of glioma for increasing (cyan) and constant (green) cell division rate.(TIFF)

Table S1
**Proto-oncogenes implicated in glioma formation.** Information on the 29 proto-oncogenes that have been implicated in the formation of glioma. The COSMIC Cancer Gene Census is a regularly-updated catalogue of somatic cell mutations causally implicated in cancer: http://cancer.sanger.ac.uk/cosmic/census. Of all genes listed, we have selected genes with a known role in glioma (including subtypes such as glioblastoma, astrocytoma, oligodendroglioma). An additional 6 genes were listed in the COSMIC gene database as being implicated in “other tumor types”. These genes, KRAS, MYC, CDKN2A(p16), CDKN2A(p14), CTNNB1(beta-catenin), and ERBB2(HER2), have indeed been implicated in gliomagenesis in other studies [43]–[46], so we have included them in this list. The probability of any one of the oncogenes being mutated is equivalent to 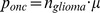, where 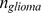 is the number of oncogenes involved in glioma formation and 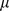 is the probability for genetic mutation due to a single cell division.(PDF)
