# Supplementary figures and images for: A Computational Model Incorporating Neural Stem Cell Dynamics Reproduces Glioma Incidence across the Lifespan in the Human Population
Source: PLoS One. 2014 Nov 19;9(11):e111219. doi: 10.1371/journal.pone.0111219 (PMC4237327; doi:10.1371/journal.pone.0111219)

**A**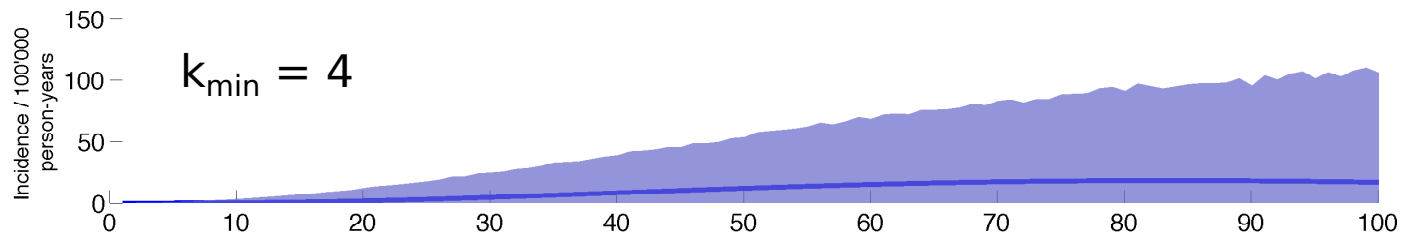**B**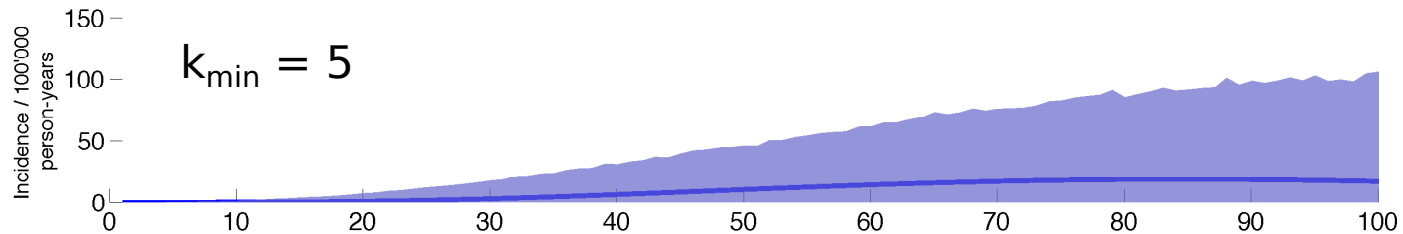**C**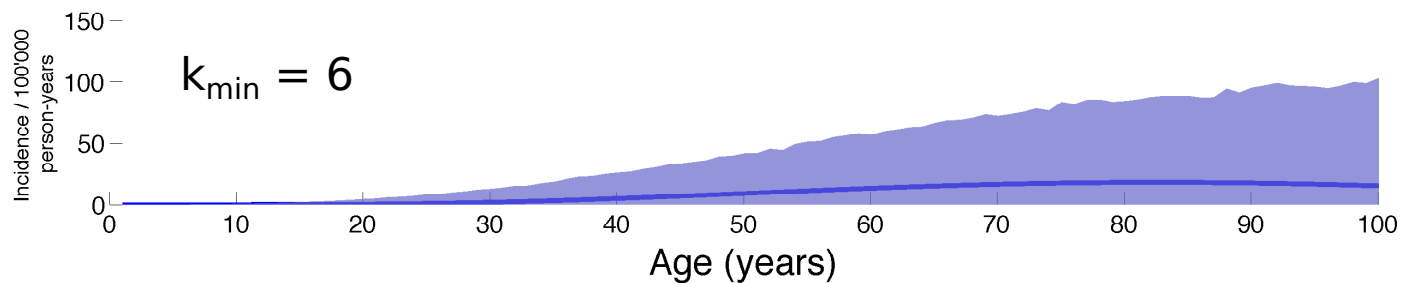

Supplement: Figure S1 — Confidence intervals for modeled incidence. 95 confidence intervals (shaded) for the modeled incidence rates during aging, as computed by bootstrapping. The modeled incidence curve (blue line) is the same as shown in Fig. 2 using (A) , and , (B) , and and (C) , and . (PDF) [file pone.0111219.s001.pdf]

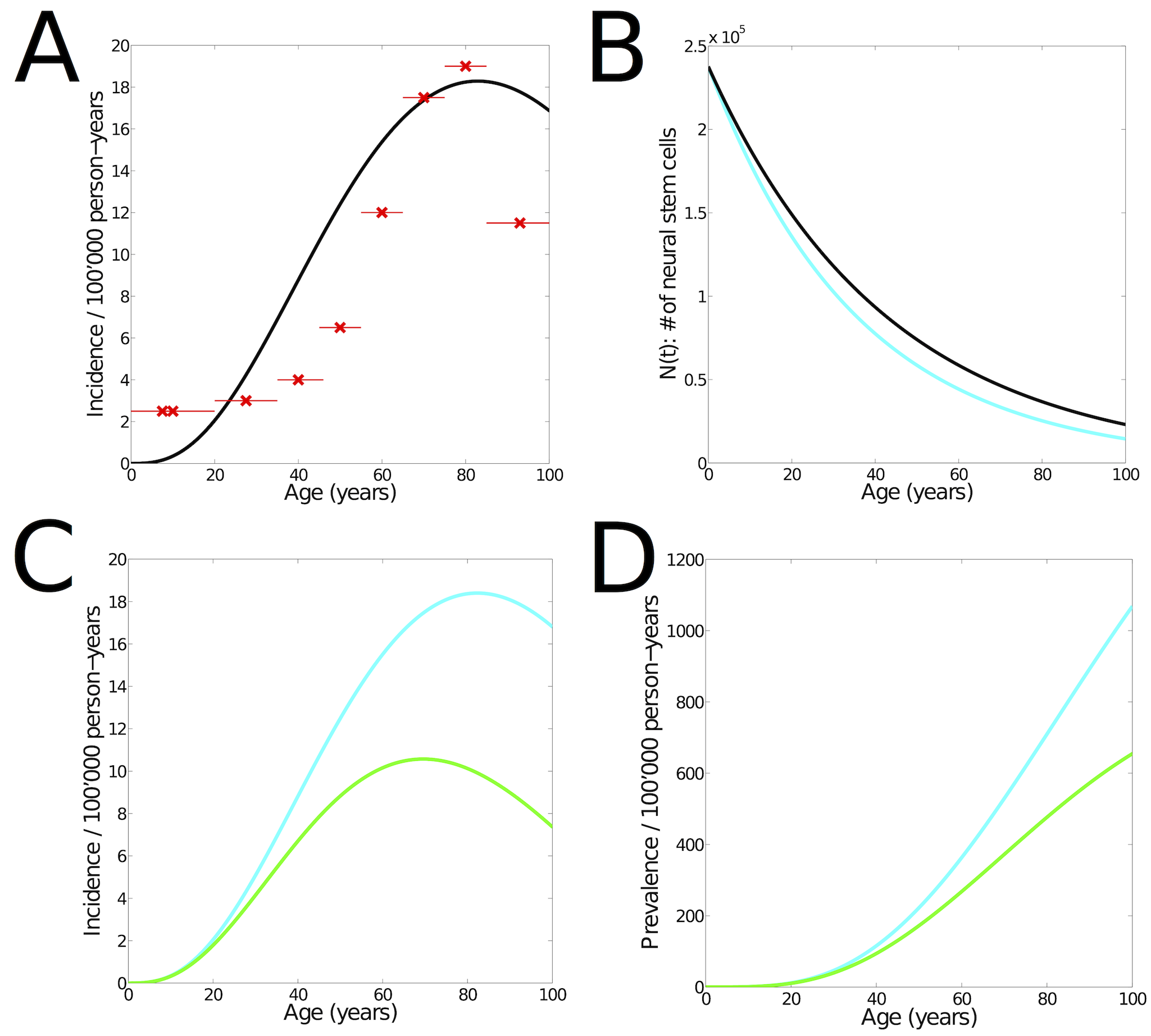

Supplement: Figure S2 — Effect of increasing cell division rate for scenario with . (A) Modeled incidence of glioma (black) under constant cell division rate (). Model parameters , and were used in order to match with the demographic data (red crosses: mean incidence of age groups, red lines: spans of age groups). The increasing proliferation rate of NSCs is therefore not a necessary condition for the incidence curve to match the demographic data, since similar results are obtained after changes in the model parameters and . (B) Number of NSCs over time, as used for the incidence curve shown in (A) (black) and for the scenario where cell division rate increases linearly (Fig. 2, cyan). Small changes in the number of NSCs over time are sufficient to make up for the constant cell division rate. It remains an empirical question which estimates of and are correct in the adult human, since these are extrapolated from the model, the young human, and the aging rodent. (C) Incidence of glioma as derived from our model, for increasing (cyan) and constant (green) cell division rate during aging. Model parameters are the same (, , ). The green curve is the predicted incidence by the model if the proliferation rate was constant, and so leads an estimate of the net effect of the increase. Overall, as for our model suggests that the increase in cell-cycle re-entry substantially increases glioma formation. (D) Prevalence of glioma for increasing (cyan) and constant (green) cell division rate. (TIFF) [file pone.0111219.s002.tiff]
