# Supplementary material for: A Computational Model Incorporating Neural Stem Cell Dynamics Reproduces Glioma Incidence across the Lifespan in the Human Population
Source: PLoS One. 2014 Nov 19;9(11):e111219. doi: 10.1371/journal.pone.0111219 (PMC4237327; doi:10.1371/journal.pone.0111219)
Supplement: Table S1 — Proto-oncogenes implicated in glioma formation. Information on the 29 proto-oncogenes that have been implicated in the formation of glioma. The COSMIC Cancer Gene Census is a regularly-updated catalogue of somatic cell mutations causally implicated in cancer: http://cancer.sanger.ac.uk/cosmic/census. Of all genes listed, we have selected genes with a known role in glioma (including subtypes such as glioblastoma, astrocytoma, oligodendroglioma). An additional 6 genes were listed in the COSMIC gene database as being implicated in “other tumor types”. These genes, KRAS, MYC, CDKN2A(p16), CDKN2A(p14), CTNNB1(beta-catenin), and ERBB2(HER2), have indeed been implicated in gliomagenesis in other studies [43]–[46], so we have included them in this list. The probability of any one of the oncogenes being mutated is equivalent to , where is the number of oncogenes involved in glioma formation and is the probability for genetic mutation due to a single cell division. (PDF) [file pone.0111219.s003.pdf]

Table S1: Proto-oncogenes implicated in glioma formation

Symbol	Name	GeneID	Chromosome	Chr Band	Tumor Types (Somatic Mutations)
APC	adenomatous polyposis of the colon gene	324	5	5q21	colorectal, pancreatic, desmoid, hepatoblastoma, glioma, other CNS
BRAF	v-raf murine sarcoma viral oncogene homolog B1	673	7	7q34	melanoma, colorectal, papillary thyroid, borderline ovarian, NSCLC, cholangiocarcinoma, pilocytic astrocytoma
CDKN2A	cyclin-dependent kinase inhibitor 2A (p16(INK4a)) gene	1029	9	9p21	melanoma, multiple other tumor types
CDKN2a(p14)	cyclin-dependent kinase inhibitor 2A– p14ARF protein	1029	9	9p21	melanoma, multiple other tumor types
CDKN2C	cyclin-dependent kinase inhibitor 2C (p18, inhibits CDK4)	1031	1	1p32	glioma, MM
CIC	capicua homolog	23152	19	19q13.2	oligodendroglioma, soft tissue sarcoma
COPEB	core promoter element binding protein (KLF6)	1316	10	10p15	prostate, glioma
CTNNB1	catenin (cadherin-associated protein), beta 1	1499	3	3p22-p21.3	colorectal, ovarian, hepatoblastoma, pleomorphic salivary gland adenoma, other tumor types
EGFR	epidermal growth factor receptor	1956	7	7p12.3-p12.1	glioma, NSCLC
ERBB2	v-erb-b2 erythroblastic leukemia viral oncogene homolog 2	2064	17	17q21.1	breast, ovarian, other tumor types, NSCLC, gastric
FUBP1	far upstream element (FUSE) binding protein 1	8880	1	1p13.1	oligodendroglioma
GOPC	golgi associated PDZ and coiled-coil motif containing	57120	6	6q21	glioblastoma
H3F3A	H3 histone, family 3A	3020	1	1q42.12	glioma
HIST1H3B	histone cluster 1, H3b	3020	6	6p22.1	glioma
IDH1	isocitrate dehydrogenase 1 (NADP+), soluble	3417	2	2q33.3	glioblastoma

Table S1 (continued). Proto-oncogenes implicated in glioma formation

Symbol	Name	GeneID	Chromosome	Chr Band	Tumor Types (Somatic Mutations)
IDH2	socitrate dehydrogenase 2 (NADP+), mitochondrial	3418	15	15q26.1	glioblastoma
KIAA1549	KIAA1549	57670	7	7q34	pilocytic astrocytoma
KRAS	v-Ki-ras2 Kirsten rat sarcoma 2 viral oncogene homolog	3845	12	12p12.1	pancreatic, colorectal, lung, thyroid, AML, other tumor types
MDM2	Mdm2 p53 binding protein homolog	4193	12	12q15	sarcoma, glioma, colorectal, other tumor types
MDM4	Mdm4 p53 binding protein homolog	4194	1	1q32	glioblastoma, bladder, retinoblastoma
MYC	v-myc myelocytomatosis viral oncogene homolog (avian)	4609	8	8q24.12-q24.13	Burkitt lymphoma, amplified in other cancers, B-CLL
NF1	neurofibromatosis type 1 gene	4763	17	17q12	neurofibroma, glioma
PIK3CA	phosphoinositide-3-kinase, catalytic, alpha polypeptide	5290	3	3q26.3	colorectal, gastric, glioblastoma, breast
PIK3R1	phosphoinositide-3-kinase, regulatory subunit 1 (alpha)	5295	5	5q13.1	glioblastoma, ovarian, colorectal
PTEN	phosphatase and tensin homolog gene	5728	10	10q23.3	glioma, prostate, endometrial
RAF1	v-raf-1 murine leukemia viral oncogene homolog 1	5894	3	3p25	pilocytic astrocytoma
ROS1	v-ros UR2 sarcoma virus oncogene homolog 1 (avian)	6098	6	6q22	glioblastoma, NSCLC
SRGAP3	SLIT-ROBO Rho GTPase activating protein 3	9901	3	3p25.3	pilocytic astrocytoma
TP53	tumor protein p53	7157	17	17p13	breast, colorectal, lung, sarcoma, adrenocortical, glioma, multiple other tumor types
